# Dopamine and the Biology of Creativity: Lessons from Parkinson’s Disease

**DOI:** 10.3389/fneur.2014.00055

**Published:** 2014-04-22

**Authors:** Eugénie Lhommée, Alina Batir, Jean-Louis Quesada, Claire Ardouin, Valérie Fraix, Eric Seigneuret, Stéphan Chabardès, Alim-Louis Benabid, Pierre Pollak, Paul Krack

**Affiliations:** ^1^U836, INSERM, Grenoble, France; ^2^University of Grenoble Alpes, GIN, Grenoble, France; ^3^Movement Disorder Unit, CHU de Grenoble, Grenoble, France; ^4^Centre d’Investigation Clinique, CHU de Grenoble, Université Joseph Fourier, Grenoble, France; ^5^Department of Neurosurgery, CHU de Grenoble, Grenoble, France

**Keywords:** STN DBS, impulse control disorders, creativity, dopamine, Parkinson’s disease

## Abstract

**Background:** Parkinson’s disease (PD) is characterized by reduced flexibility, conceptualization, and visuo-spatial abilities. Although these are essential to creativity, case studies show emergence of creativity during PD. Knowledge about the role of dopamine in creativity so far only stems from a few case reports. We aim at demonstrating that creativity can be induced by dopaminergic treatments in PD, and tends to disappear after withdrawal of dopamine agonists.

**Methods:** Eleven consecutive creative PD patients were selected from candidates for subthalamic nucleus deep brain stimulation (STN DBS) surgery, and compared to 22 non-creative control PD patients. Motor disability (UPDRS III), cognition (Frontal score, Mattis scale), and behavior (Ardouin scale) were assessed before surgery and 1 year after.

**Results:** Before surgery, whereas cognitive and motor assessments were similar between groups, dopamine agonist (but not levodopa) dosages were higher in creative patients (*p* = 0.01). The Ardouin scale revealed also a specific psycho-behavioral profile of creative patients which had higher scores for mania (*p* < 0.001), hobbyism (*p* = 0.001), nocturnal hyperactivity (*p* = 0.041), appetitive functioning (*p* = 0.003), and ON euphoria (*p* = 0.007) and lower scores for apathy and OFF dysphoria (*p* = 0.04 for each). Post-operative motor, cognitive, and behavioral scores as dopaminergic treatment dosages were equivalent between groups. Motor improvement allowed for a 68.6% decrease in dopaminergic treatment. Only 1 of the 11 patients remained creative after surgery. Reduction of dopamine agonist was significantly correlated to the decrease in creativity in the whole population of study (Spearman correlation coefficient ρ = 0.47 with confidence index of 95% = 0.16; 0.70, *p* = 0.0053).

**Conclusion:** Creativity in PD is linked to dopamine agonist therapy, and tends to disappear after STN DBS in parallel to reduction of dopamine agonists, which are relatively selective for the mesolimbic D3 dopamine receptors.

## Introduction

For a production to qualify as “creative,” it must fulfill two criteria: it must be new (or original) and useful (or valuable, or relevant) (1). However, this both criterions are subjective. A creative work is not the result of a single cognitive process, of neuronal activity in one isolated cerebral area, or of a unique mental faculty, but of the interaction of multiple cognitive processes supported by a large network of multiple cerebral areas. The neurotransmitter dopamine plays a crucial role in this complex interaction. A link seems to exist between mental illness, notably bipolar disorder, dopamine, and creativity. Many well-known artists (e.g., Edward Munch, Ernest Hemingway, and Virginia Woolf) suffered from bipolar disorder and 38.3% of British artists who received awards were treated for affective disorders (2). A strong dopaminergic component is reported in bipolar disorder, treated by antidopaminergic medication. Many artists admit to being more creative under the influence of psychoactive or “psychedelic” drugs. Drugs that can lead to addiction induce a large release of dopamine in the mesolimbic pathway, directly for amphetamine and cocaine by blocking dopamine reuptake, and indirectly for other psychoactive drugs such as alcohol (3). The dopaminergic mesolimbic pathway is involved in the control of motivational, reward, and reinforcement processes and contributes to dependence and addiction. Eysenck proposed that people in whom the “psychoticism” personality trait is strong, experience a “widening of the associative horizon,” which could generate creativity by weakening latent inhibition of thought (4). He also reported a link between psychoticism, which is highly represented in mental illness and increased dopamine levels. Flaherty proposed a tridimensional model of creativity, in which frontal, temporal, and dopaminergic systems modulate idea generation and creativity, influenced by medical conditions and treatments (5, 6). Two recent neurobiological studies established a link between dopamine and creativity (7, 8). In Parkinson’s disease (PD), (i) depletion of the substantia nigra and the ventral tegmental area causes low dopamine concentration in the brain and (ii) cognitive impairment diminishes flexibility processes essential to creativity. To Lakke’s surprise, artistic activity persisted in PD, and in the case of several patients was awakened, from the time of disease onset (9). Many case reports confirm exacerbation or revelation of creative art work in PD patients treated by dopamine replacement therapy (DRT) and especially dopamine agonists (10–13). Very thoughtfully and exhaustive reviews sustain strongly the link between dopaminergic treatment and awakening of creativity in PD, but to date, there is only case report and no group study permitting to support this assertion (6, 14). Deep brain stimulation (DBS) of the subthalamic nucleus (STN) allows reduction of DRT by 50% on average, maintaining a stable and relatively good motor state (15). STN stimulation in PD allows longitudinal monitoring of the consequences of dopamine modulation on creativity and other motivational behaviors (16). DBS targets the sensorimotor part of the STN, which depends on the nigrostriatal dopaminergic pathway. The limbic part of the STN, which depends on the mesolimbic dopaminergic pathway, is less influenced by DBS. STN DBS thus can indirectly unmask non-motor symptoms related to lesions in the mesolimbic dopaminergic system (17). We conducted a case controlled comparative study in PD patients undergoing STN DBS surgery to assess the influence of DRT on creativity. By comparing psychological features in creative and non-creative patients, we aimed to describe the potential individual co-variables of creativity, particularly hypomanic mood and impulse control disorders, which are also modulated by DRT.

## Materials and Methods

### Study population and design

A total of 76 consecutive patients underwent bilateral STN DBS in Grenoble between December 2004 and August 2007. Selection criteria for surgery were: (i) clinically diagnosed PD; (ii) severe l-DOPA-related motor complications despite optimal adjustment of anti-Parkinsonian medication; (iii) age under 70 years; and (iv) absence of surgical contraindications, dementia, or major on-going psychiatric illness (18). Eleven patients fulfilled the study’s specific inclusion criteria, i.e., had a score ≥2 for the “creativity” item of the Ardouin scale (16, 19). For each identified creative case, two PD controls with no significant creative behavior (“creativity” ≤1 on the Ardouin scale) were selected. These PD controls had undergone STN DBS surgery immediately before and immediately after the identified case. The sample’s characteristics are described in Table 1. Assessments took place in the month preceding surgery and 1 year (±1 month) later. Exhaustive evaluations of mood, behavior, and cognition were carried out during routine hospital visits by a clinical neuropsychologist experienced in the assessment of neuropsychiatric symptoms in PD. All evaluations were conducted prospectively; data collection was retrospective. We added a case report to illustrate the changes in creative behavior related to dopamine agonist modifications.

**Table 1:** **Patients’ characteristics before and 1 year after surgery, expressed by median (25th; 75th percentile)**.

	Before surgery			1 year after surgery		
	Creative group (*n* = 11)	Control group (*n* = 22)	*p*	Creative group (*n* = 11)	Control group (*n* = 22)	*p*

**GENERAL CHARACTERISTICS**						
Sex (% of female)	45.5%	31.8%	0.443	–	–	
Age (years)	53 (51; 57)	56.5 (52; 63)	0.358	–	–	
Disease duration (years)	11 (9; 12)	11 (9; 14)	0.758	–	–	
Hemi body onset of PD (% of L/R/Bilat.)	(45/50/5)	(55/45/0)				
DRT treatment duration (years)	10 (7; 12)	9.5 (7; 13)	0.673	–	–	
Education (years)	12 (9; 17)	9 (9; 14)	0.160	–	–	
**DRT**						
Dopamine agonist equivalent dose (mg/day)	400 (350; 500)	300 (180; 320)	0.012	120 (0; 210)	37.5 (0; 400)	0.952
l-DOPA (mg/day)	885 (450; 1170)	1070 (845; 1320)	0.181	100 (0; 300)	75 (0; 325)	0.830
Total dopamine equivalent dose (mg/day)	1980 (1400; 2760)	2440 (1920; 2950)	0.359	300 (150; 900)	575 (150; 900)	0.970
**MOTOR OUTCOME**						
UPDRS III ON medication /108	9.5 (8; 12)	8.5 (5; 10.5)	0.130	8 (5; 17.5)	10.8 (6.5; 18)	0.359
UPDRS III OFF medication /108	36 (33; 41)	36 (28; 43)	0.909	11 (8; 28)	15.5 (11.5; 25)	0.422
**COGNITIVE OUTCOME**						
Mattis dementia rating scale /144	141 (140; 143)	137.5 (132; 140)	0.018	141 (138; 142)	136.5 (130; 140)	0.043
Attention /37	36 (35; 37)	35.5 (35; 36)	0.303	36 (35; 37)	35.5 (35; 36)	0.440
Initiation /37	37 (37; 37)	35.5 (34; 37)	0.022	37 (35; 37)	33.5 (29; 37)	0.022
Construction /6	6 (6; 6)	6 (6; 6)	0.480	6 (6; 6)	6 (6; 6)	1.000
Conceptualization /39	38 (36; 39)	37 (36; 38)	0.329	39 (38; 39)	37.5 (34; 39)	0.077
Memory /25	24 (24; 25)	24 (22; 25)	0.443	24 (23; 25)	25 (23; 25)	0.566
Frontal score /50	41.9 (41; 46.6)	37.3 (29.5; 43.5)	0.136	41.8 (40.3; 44.7)	39.5 (29.8; 45)	0.340
Wisconsin Card Sorting Test /20	15 (15; 20)	12 (9; 18)	0.085	18 (15; 20)	16.5 (9; 18)	0.082
Verbal fluency /10	8.6 (7.7; 10)	8.3 (7; 10)	0.493	6.7 (6.3; 8.3)	7 (5.3; 9)	0.939
Motor series /10	10 (9.2; 10)	9.3 (6.1; 10)	0.076	8.6 (7.4; 10)	9.3 (6.8; 10)	0.937
Graphic series /10	7 (6.2; 10)	7 (5; 10)	0.433	10 (6.2; 10)	8.8 (5; 10)	0.434
**APATHY AND DEPRESSION**						
Beck depression inventory /63	6 (1; 8)	10 (8; 17)	0.002	8 (6; 9)	6.5 (3; 11)	0.455
Starkstein apathy scale /42	5 (2; 7)	8.5 (5; 12)	0.021	10 (7; 13)	14 (10; 17)	0.283

*After surgery, evaluations are made under chronic stimulation parameters*.

### Outcome measures

#### Motor outcome and treatment

Dopaminergic treatment was expressed in (i) daily dopamine agonist equivalent dose, (ii) daily l-DOPA dose, and (iii) total daily dopamine equivalent dose. Dopamine agonist equivalent dose was calculated by comparison to 100 mg l-DOPA in terms of motor anti-Parkinsonian effect (18). Total daily dopamine equivalent dose is the sum of dopamine agonist equivalent and l-DOPA doses. Psychotropic medication (atypical neuroleptic, antidepressant, benzodiazepine, sleeping pills) was noted. Chronic stimulation parameters were noted at post-operative follow-up. The Unified Parkinson’s Disease Rating Scale part III (motor score) was used to assess the beneficial effects of l-DOPA and subthalamic stimulation on Parkinsonian motor signs (20). Before surgery, UPDRS III was evaluated in OFF and ON medication conditions using suprathreshold doses of l-DOPA (18). This assessment was repeated at follow-up, in four treatment conditions (OFF medication/ON stimulation; OFF medication/OFF stimulation; ON medication/OFF stimulation; ON medication/ON stimulation).

#### Cognitive evaluation

Overall cognitive function was assessed using the Mattis dementia rating scale (21). The degree of frontal–subcortical deterioration was evaluated using the frontal score, a more specific test battery measuring frontal executive function (22). Assessment included the simplified version of the Wisconsin Card Sorting Test (23), verbal fluency tests (24), and graphic and motor series (25).

#### Evaluation of mood and behavior

##### Ardouin scale

This previously described instrument (19) is currently undergoing validation in PD (Rieu et al., submitted). Patients’ general psychological state is assessed (depressive, hypomanic or manic mood, anxiety, irritability, hyper-emotivity, and psychotic symptomatology), as are apathy, non-motor fluctuations (non-motor ON and non-motor OFF), and hyperdopaminergic behaviors (12 items: nocturnal hyperactivity, diurnal somnolence, excessive eating, creativity, hobbyism, punding, risk-seeking behavior, compulsive shopping, pathological gambling, hypersexuality, compulsive dopaminergic medication use, and overall functioning in an appetitive mode). The frequency and intensity of a symptom’s occurrence in the preceding month is rated on a scale ranging from 0 (absent) to 4 (severe). A score of 0 indicates no modification of the patient’s usual habits; a score of 1 reflects slight modification; a score of 2 is indicative of a moderate modification in habitual behavior that is usually significant enough to require therapeutic adjustment; and a score >2 equates with clear-cut maladaptive pathological behavior requiring immediate therapeutic intervention. All 11 creative patients scored ≥2 on the “creativity” item, and had experienced a recent emergence of or increase in creative activity, accompanied by an addictive driving-force (e.g., individuals pursued the creative activity for longer than initially intended, especially at night; their daily activity revolved around creativity, and considerable time was devoted to it; other behavioral repertoires, such as meals, social, occupational, and recreational activities were abandoned in favor of the creative activity, as were family or professional obligations).

##### Beck depression inventory II

The Beck depression inventory (BDI), a self-reported scale validated in PD, was used to determine the severity of depressive symptoms (26, 27).

##### Starkstein apathy scale

Since a lack of motivation is often observed in the post-operative year following STN DBS (17), and probably influences the desire to engage in creative activity, the Starkstein apathy scale was used to measure motivation (28, 29).

#### Statistical analyses

Categorical parameters were summarized in terms of size and frequency, and continuous parameters by median and 25th; 75th percentiles. A Mann–Whitney test was performed on all parameters, in creative and control groups before surgery and 1 year after surgery. A Wilcoxon test was used to compare each of the all variables before surgery and 1 year after, in both groups. Independence between qualitative parameters was assessed using the chi-square test. A non-parametric Spearman test was completed to evaluate rank correlation coefficient. *p*-Values <0.05 were considered statistically significant. Statistical analyses were performed using STATA release 12 (StataCorp, College Station, TX, USA) PC-software.

## Results

The general characteristics of the patients are shown in Table 1.

In creative patients, artistic work either started when taking DRT (*n* = 6) or pre-existing creativity was markedly exacerbated after the introduction of DRT (*n* = 5). Art work consisted of sculpting (*n* = 1), face casting (*n* = 1), painting (*n* = 3), glass painting (*n* = 1), drawing (*n* = 1), graphic design (*n* = 1), and writing (poetry *n* = 1, history book *n* = 1, short stories *n* = 1). Two patients in the control group had a “creativity” item score = 1, indicating a slight recent emergence or exacerbation of creative activity.

While creative and control patients’ total DRT dosages were the same, creative patients’ dopamine agonist equivalent doses were higher than controls’ at baseline (Table 1). Motor improvement permitted post-operative reduction in dopamine agonist equivalent and total DRT equivalent doses to the same extent in both groups, i.e., by 68.6% on average: l-DOPA daily dose was reduced from 885 to 100 mg/day (*p* = 0.008) in the creative group and from 1070 to 75 mg/day in the control group (*p* ≤ 0.001); similarly, daily dopamine agonist equivalent dose was reduced from 400 to 120 mg/day in the creative group (*p* = 0.014) and from 300 to 37.5 mg/day in the control group (*p* = 0.240). Reduction of dopamine agonist was significantly correlated to reduction of creativity in the whole population of study [Spearman correlation coefficient ρ = 0.47 with confidence index of 95% = 0.16; 0.70, *p* = 0.0053].

Eleven patients in the creative group were taking dopamine agonists at preoperative assessment, 6/11 were treated by ropinirole, 3/11 by piribedil, 1/11 by pramipexole, and 1/11 by pergolide. One patient in the creative group was taking amantadine. Eighteen of 22 patients in the control group were taking dopamine agonists at preoperative assessment, 4/22 were treated by ropinirole, 5/22 by piribedil, 3/22 by pramipexole, and 6/22 by pergolide. Five patients in the control group were taking amantadine. Thirty-three patients were treated by l-DOPA before surgery. At post-operative assessment, 7/11 creative patients were treated by l-DOPA, and 14/22 control patients. Seven of 11 creative patients were treated by dopamine agonists (3/7 ropinirole, 2/7 piribedil, and 2/7 pramipexole) vs. 10/22 control patients (1/10 ropinirole, 8/10 piribedil, 1/10 bromocriptine). Median dosages are presented in Table 1. Stimulation parameters did not differ between groups and were similar for both hemispheres, with a mean (±SD) stimulation strength of 2.9 ± 0.4 V in creative patients vs. 3.1 ± 0.4 in control patients, a median (25th–75th) frequency of 130 (130–145) Hz in the creative group vs. 130 (130–145) in the control group, and a median (25th–75th) pulse duration of 60 (60–60) μs in the creative group vs. 60 (60–75) in the control group. In each group, one patient was on clozapine, an atypical neuroleptic (Table 2). Creative patients were more frequently treated by antidepressants and benzodiazepines than control patients from baseline (Table 2). The small sample size does not, however, permit statistical analysis.

**Table 2:** **Psychotropic sedative drugs**.

	Before surgery	1 Year after surgery
Creative patients (*n* = 11)	Neuroleptic: *n* = 1	Neuroleptic: *n* = 1
	Antidepressant: *n* = 4	Antidepressant: *n* = 3
	Benzodiazepine: *n* = 4	Benzodiazepine: *n* = 4
	Soporific: *n* = 0	Soporific: *n* = 0
Control patients (*n* = 22)	Neuroleptic: *n* = 1	Neuroleptic: *n* = 1
	Antidepressant: *n* = 1	Antidepressant: *n* = 2
	Benzodiazepine: *n* = 0	Benzodiazepine: *n* = 2
	Soporific: *n* = 2	Soporific: *n* = 0

At preoperative and post-operative assessments, ON and OFF medication conditions for UPDRS motor scores did not differ between groups (Table 1). Post-operative improvement in UPDRS motor score in off drug on stimulation condition was superior by 50% on average.

Overall cognitive function performance was better in creative patients than in controls, with higher scores in the “initiation” subscale of the Mattis dementia rating scale, which measures verbal fluency and graphic automatisms (Table 1). There was no difference in executive function scores in the two groups. Verbal fluency decreased in all patients following surgery: median scores went from 8.6/10 to 6.7/10 in creative patients (*p* = 0.029) and from 8.3/10 to 7/10 in control patients (*p* = 0.003).

Before surgery, creative patients exhibited a specific pattern of hyperdopaminergic behaviors at the Ardouin scale (Table 3): they were more hypomanic, more active at night, had higher scores on “hobbyism,” and functioned in a more markedly appetitive mode than control patients. They were less apathetic. They had more pronounced non-motor ONs phases (they experienced greater artificial euphoria during ON phases), and less severe non-motor OFFs phases (they were less dysphoric, anxious, tired during OFF phases). One year after surgery, overall differences between creative and control patients had diminished: creativity and appetitive functioning were still more highly represented in the creative group, but there were no longer any differences in the other variables. One year after surgery, following the reduction in dopaminergic treatment, creative patients were less hypomanic (*p* = 0.004), more apathetic (*p* = 0.016), non-motor ON had diminished (*p* = 0.003) as had nocturnal hyperactivity (*p* = 0.015), creativity (*p* = 0.002), hobbyism (*p* = 0.004), and appetitive functioning (*p* = 0.003). Hyperdopaminergic behaviors also diminished in control patients. One year after surgery, clinically relevant creative behavior persisted in only 1 of the 11 patients.

**Table 3:** **Ardouin scale scores expressed by median (25th; 75th percentile) before and 1 year after surgery**.

	Before surgery			1 year after surgery		
	Creative group (*n* = 11)	Control group (*n* = 22)	*p*	Creative group (*n* = 11)	Control group (*n* = 22)	*p*

**GENERAL PSYCHOLOGICAL STATE**						
Depressive mood	0 (0; 1)	0 (0; 1)	0.883	0 (0; 1)	0 (0; 1)	0.795
Hypomanic or manic mood	1 (1; 2)	0 (0; 0)	≤0.001	0 (0; 0)	0 (0; 0)	1.000
Anxiety	0 (0; 1)	0 (0; 1)	0.810	0 (0; 0)	0 (0; 1)	0.136
Irritability	0 (0; 1)	0 (0; 1)	0.760	0 (0; 1)	0 (0; 0)	0.647
Hyper-emotivity	0 (0; 1)	1 (0; 2)	0.196	0 (0; 0)	0.5 (0; 1)	0.057
Psychotic symptomatology	0 (0; 0)	0 (0; 1)	0.373	0 (0; 0)	0 (0; 0)	0.437
**OVERAL FUNCTIONING IN APATHETIC MODE**						
Apathy	0 (0; 0)	0 (0; 1)	0.040	1 (0; 2)	0.5 (0; 2)	0.759
**NON-MOTOR FLUCTUATIONS**						
Non-motor ON	2 (2; 3)	1 (0; 2)	0.007	0 (0; 0)	0 (0; 0)	0.310
Non-motor OFF	0 (0; 2)	1 (1; 3)	0.040	0 (0; 0)	0 (0; 1)	0.185
**HYPERDOPAMINERGIC BEHAVIOURS**						
Nocturnal hyperactivity	1 (0; 3)	0 (0; 0)	0.041	0 (0; 0)	0 (0; 0)	0.480
Diurnal somnolence	0 (0; 1)	0.5 (0; 1)	0.558	0 (0; 0)	0 (0; 1)	0.463
Excessive eating behaviour	1 (0; 1)	0 (0; 2)	0.228	1 (1; 2)	0 (0; 1)	0.008
Creativity	2 (2; 3)	0 (0; 0)	≤0.001	0 (0; 1)	0 (0; 0)	0.003
Hobbyism	3 (1; 3)	0 (0; 1)	0.001	0 (0; 0)	0 (0; 0)	0.157
Punding	0 (0; 0)	0 (0; 0)	0.611	0 (0; 0)	0 (0; 0)	1
Risk-seeking behaviour	0 (0; 1)	0 (0; 0)	0.538	0 (0; 1)	0 (0; 0)	0.012
Compulsive shopping	0 (0; 1)	0 (0; 0)	0.574	0 (0; 0)	0 (0; 0)	0.480
Pathological gambling	0 (0; 0)	0 (0; 0)	0.206	0 (0; 0)	0 (0; 0)	0.480
Hypersexuality	0 (0; 1)	0 (0; 1)	0.653	0 (0; 0)	0 (0; 0)	0.206
Compulsive dopaminergic medication use	1 (0; 2)	0 (0; 2)	0.493	0 (0; 0)	0 (0; 0)	0.710
Overall functioning in an appetitive mode	2 (2; 3)	1 (0; 1)	0.003	0 (0; 1)	0 (0; 0)	0.005

Before surgery, creative patients had lower scores for depressive mood than control patients (*p* = 0.002) at the BDI. After surgery, there was no difference between groups. Before surgery, creative patients were less apathetic than control patients (*p* = 0.021). After surgery, apathy scores increased in both groups: from 5 to 10 in creative patients (*p* = 0.003) and from 8.5 to 14 in control patients (*p* = 0.049) (Table 1).

## Case Report

**Figure 1:**
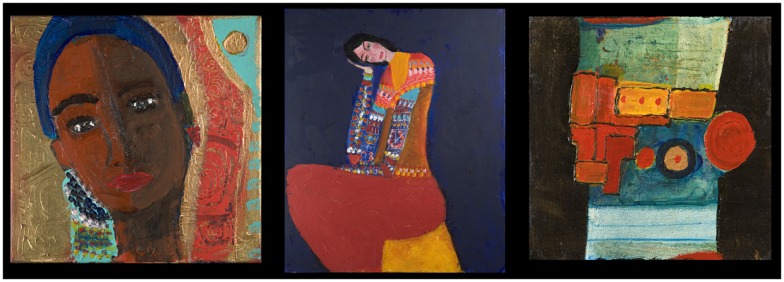
**Three works from her first, non-addictive period**.

**Figure 2:**
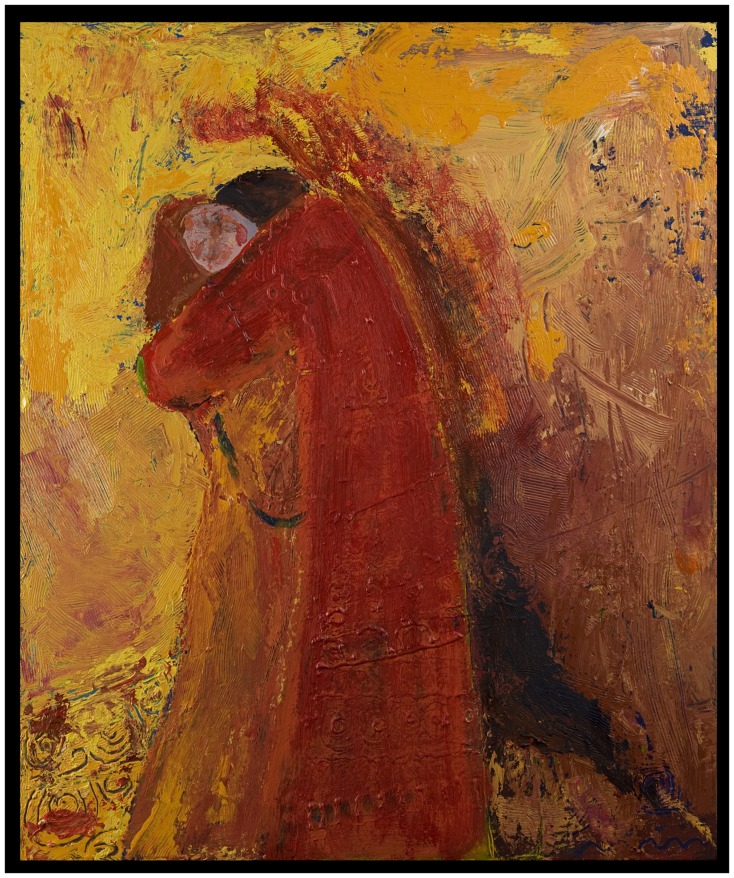
**Addictive period**. Painting of romantic relationships illustrating the change in content and technique.

**Figure 3:**
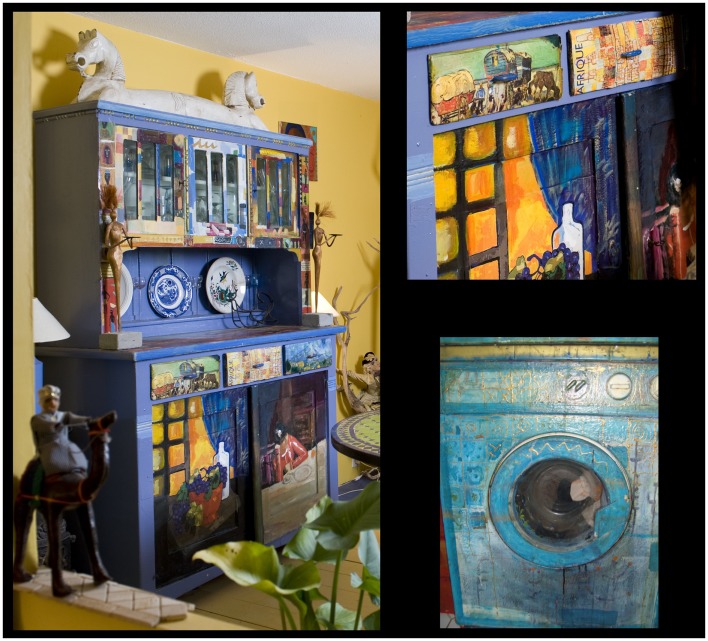
**Addictive period**. Examples of furniture and equipment being transformed in pieces of art illustrating the change in quantity and in surfaces of application.

**Figure 4:**
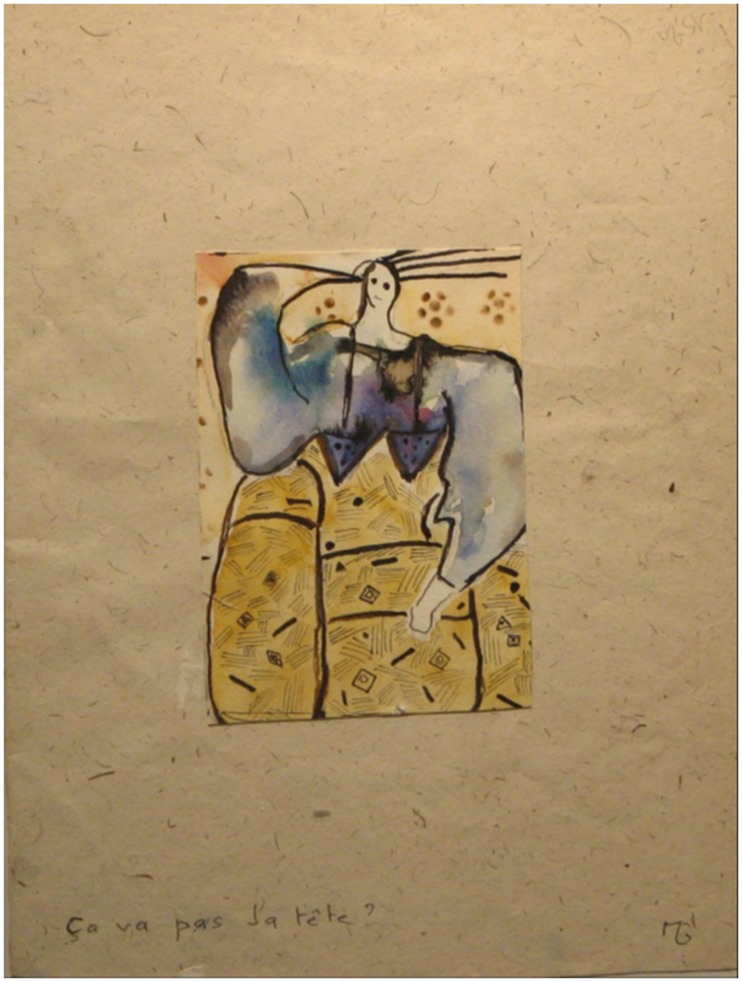
**Addictive period**. The painting bears the inscription “Am I going nuts?” illustrating maintenance of self-criticism by the patient.

**Figure 5:**
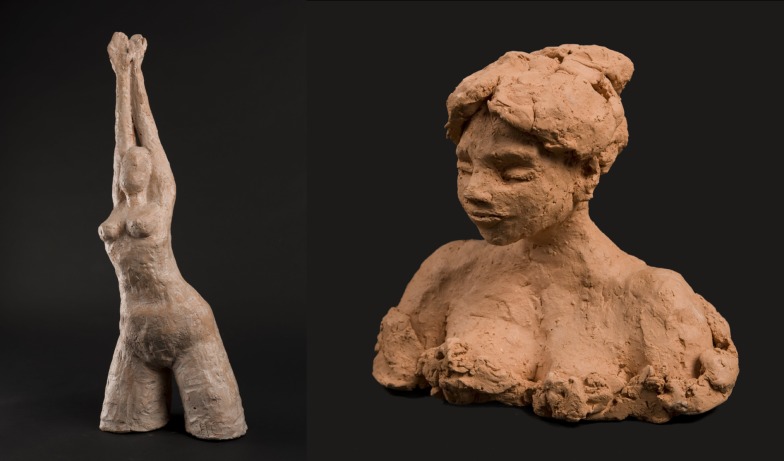
**Resumption of a calm and satisfying creativity**.

The case of this woman illustrates the influence of dopamine agonists on creativity. PD started in 1994, at age 41, with pain in the right arm. She developed a depressive syndrome on learning the PD diagnosis. A few years later, on a high dose of pramipexole (2.8 mg/day) associated with levodopa (daily equivalent dose of levodopa = 1100 mg/day), she experienced strong exacerbation of her painting activity (Figure 1), accompanied by nocturnal hyperactivity and psychosis. Dopaminergic addiction followed, as did an escalation to painting addiction (Figures 2–4), associated with compulsive buying of painting material and risk-taking behavior. Her life-style changed completely, and her home became a gathering place where her artist friends met up and partied. All the behavioral modifications she experienced had major repercussions, upsetting her personal, family, and social equilibrium, and led to hospitalization, reduction in dopaminergic treatment, and the introduction of clozapine. A subsequent increase in akinesia required higher doses of levodopa, which in turn induced the onset of motor complications. As a result, STN DBS surgery was performed in 2009. Neurosurgical treatment led to improvement in motor fluctuations and reduction of dopaminergic therapy. Her creative activity remained rich, and was judged by the patient herself to be more tranquil and satisfying. She mostly sculpts now (Figure 5). She did not dare to pick up a paintbrush for a long time. She was afraid of falling prey to the devastating addiction to painting again. Here is her own account: I’ve always drawn and painted. As an adolescent, I would paint on the walls of my attic. But in 2002, I embraced painting totally (Figure 1). I transformed my home into a studio, with tables and canvases everywhere. I was so happy. My illness got worse in 2004: I stopped working and went on new medication. At that point, I started painting from morning till night, and often all through the night until morning. I was obsessed with painting. bought huge amounts of materials, and used countless numbers of brushes at a time. I used knives, forks, sponges … I would gouge open tubes of paint – it was everywhere (Figure 2) … But I was still in control at that point. Then, the urge to paint became incontrollable. I started painting on the walls, the furniture, even the washing machine (Figure 3). I would paint any surface I came across. I also had my “expression wall” and I could not stop myself from painting and repainting this wall every night in a trance-like state. My uncontrollable creativity had turned into something destructive (Figure 4). My partner could no longer bear it. People close to me realized that I crossed some kind of line into the pathological, and in 2006, at their instigation, I was hospitalized. Today, my doctors have succeeded in getting my medication under control, and my creativity has become more tranquil and structured. It has once again become a pleasure, which upsets no-one (Figure 5) (30).

Works illustrating the periods of the patient’s creativity are reproduced with her authorization (Figures 1–5).

## Discussion

### Frequency of creativity

Our study revealed a high prevalence of creativity (14.5%). This contrasts with the small number of cases reported in the literature to date. The scale used probably enabled easier detection of creativity than do patients’ spontaneous accounts of this aspect of their lives, as it systematically evaluates all modifications in behavior. Moreover, surgical candidates are, typically, young, have long disease and treatment durations, and severe motor symptoms justifying high doses of DRT including dopamine agonists which are not recommended for the elderly (31). Hyperdopaminergic behavioral modifications are frequently and commonly observed in this Parkinsonian sub-population. In another, larger cohort, prevalence of creativity was quite similar (16).

### Creativity and punding

Punding is a frequent behavioral modification in PD, in which prevalence ranges from 0.3 to 14% and is associated with dopaminergic medications and impulse control disorder (32). Punding describes a heterogeneous set of aimless, stereotyped behaviors performed for long periods of time at the expense of other activities (33). To our point of view, creativity is the opposite of punding in its fundamental characteristics: by definition, creativity implies: (i) novelty, not repetitiveness and (ii) usefulness, not aimlessness. In fact, punding is even discussed to be included in the rubric of stereotypies (34).

### Link between creativity and dopamine agonists

Reduction of creativity was correlated with decrease in dopamine agonists’ drugs. Creative patients took higher doses of dopamine agonists than control patients before surgery. Both groups were, however, similar in terms of severity of motor symptomatology, and stage of disease. It seems that, as has been demonstrated for other behavioral modifications, dopaminergic agonists play a crucial role in the occurrence of addictive appetence to pleasure, whether it concerns creative behavior, gambling, shopping, eating, or sexual activity (35). Obviously, creativity belongs to activities driven by the reward system.

Because of their high affinity with D3 receptors which are mainly expressed in the mesolimbic pathway, like psychedelic drugs, currently available D2–D3 dopamine agonists may facilitate creative ideas and their expression. It seems plausible that dopamine agonists encourage greater freedom of association and artistic production.

Artists such as Jack Kerouac, Jean-Paul Sartre, Johnny Cash, or Andy Wharhol, who all used amphetamines to facilitate creative inspiration in their specific artistic domains can be cited to illustrate our point of view. Jack Kerouac typed his best-seller “*On the road*” in 3 weeks, working day and night, on a continuous roll of paper, chosen to avoid interrupting his rhythm, in a creative “trance” that he could not bear to break by changing the paper in his typewriter. Amphetamines induce an acute increase in dopamine in the accumbens shell (36), which expresses the D3 receptor (37). While pharmacological, neuro-anatomical, and genetic data suggest a crucial role of the D3 receptor in pharmaco-dependence (38), it would also be interesting to study its implication in behavioral addictions which seem to have a mechanism in common with drug addiction (16).

Dopamine agonists can induce hallucinations in which static objects change into moving or living objects. They are therefore likely to alter perception and perhaps facilitate visual inspiration (39).

Moreover, by acting on the nucleus accumbens, one of the main components of cerebral reward circuits (40), dopamine agonists probably encourage positive feedback on the creative artist’s own work, which makes presenting his/her work to others easier due to increased self-confidence, audacity, and non-conformism. This is compatible with the individual psychological predispositions of our creative patients, who were indeed more hypomanic than control patients.

Finally, mesolimbic denervation leads, via compensatory mechanisms, to increased sensitivity to the psychostimulant effects of dopaminergic treatment, as has been shown in the animal model of dopaminergic lesions treated with dopaminergic drugs (41, 42). This is analogous with the higher propensity of PD patients with more severe nigrostriatal denervation to develop dyskinesia (43).

### Creativity reversal after reduction of dopaminergic therapy

We demonstrated indirectly that creativity is at least partly dopamine dependent, since it diminished significantly following reduction in dopaminergic treatment permitted by STN stimulation. Only 1 of our 11 creative patients continued to maintain a high level of creativity 1 year after surgery. Patients who immerse themselves in creativity are typically convinced that their passion is the expression of their own personality and not influenced by the drugs they are taking to treat Parkinsonism. They are attached to their creativity, since it is a source of strong personal enrichment, an “awakening” that is socially recognized (unless it escalates to addictive pathological behavior, as in our case study). When surgery is envisaged by medical staff, creative patients should be warned that it could, as observed in this study, induce a decrease in creative activity via the reduction in dopaminergic treatment. If treatment is maintained, the cumulative effects of stimulation and dopamine agonists could exacerbate creativity (44), and other hyperdopaminergic behaviors (45). The creativity–dopamine agonist therapy link and the absence of difference between both hemisphere parameters of STN DBS we found do not support previous work showing an acute and lateralized direct annihilating effect of STN DBS on creativity in an isolated PD patient (46). Reduction in DRT, especially dopamine agonists, permitted by STN DBS induces not only a decrease in creativity, but also an overall decrease in motivated behaviors (16) reaching severe apathy level (17). Dopaminergic treatment seems to influence all goal-directed behaviors with a reward component, contrary to the disease itself which leads to loss of motivation related to mesolimbic dopaminergic denervation (17). This explains the continuum of observed behaviors in PD with awakening of the desire to undertake pleasant activities if the mesolimbic system is rich in dopamine, and extinction of the desire to undertake pleasant activities if the mesolimbic system lacks dopamine.

### Psychological profile of creative patients

Creative patients are more hypomanic, more active at night; they pursue more other hobbies and function in a more appetitive mode than controls. They are also less apathetic and experience more pronounced ON euphoria and less severe OFF dysphoria. This profile corresponds to an enthusiastic and energetic personality, with a proliferation of ideas and desires, an ease of association, a less conventional way of thinking, good levels of self-confidence, and belief in their capacity to accomplish great things. We suggest that there may be a link between the high prevalence of creativity in PD patients with a hypomanic profile and bipolar disorder which is also associated with creativity (47). We wonder if it would be legitimate to make an analogy between bipolar disorder and fluctuating PD patients, who are subject to repeated, ultra-rapid mood cycles parallel to motor fluctuations, in the same day. Creative patients in our study were taking more dopamine agonists and antidepressant and anxiolytic drugs. Could their medication actually cover up more severe non-motor OFFs requiring higher doses and association of medication than those of control patients? PD and bipolar disorder both lead to over-expression of creative talent, favoring the hypothesis that mesolimbic dopaminergic system dysfunction is a mechanism of bipolar disorder as it is the case in PD.

Creative patients did not necessarily present more impulse control disorders or l-DOPA addiction than controls, on the contrary, although they have higher doses of dopamine agonist. This result is compatible with the absence of association between artistic creativity and impulsivity or impulse control disorder observed by Canesi et al. (48), in contrast to other work showing that involvement in a creative or artistic profession can be a potential risk to develop impulse control disorder in PD (49).

### Other factors explaining creativity

Given the importance of a well-functioning prefrontal cortex for creativity exist, it is conceivable that the bilateral insertion into the frontal lobe of microelectrodes and DBS leads has an impact on creativity. This would count for cases as well as controls, but since the controls were already not creative before surgery, decreased creativity would not be evident in this group. However, our data showed a good maintain of frontal score (superior to 40/50, which is good) both in cases and controls after surgery.

When examining the influence of the side of the body onset of PD, we did not find any association with creativity, on the contrary to previous work (50).

Dopamine agonists probably facilitate creativity, but do not generate it on their own. Non-neurobiological factors favoring creativity in PD should also be investigated in order to advance research in this domain. PD patients need to communicate by unconventional means of expression in order to face up to their pathology which is often difficult for their relatives to understand, and socially stigmatized. Onset of PD generally occurs around retirement age and Parkinson-related disability may also lead to premature discontinuation of professional activity. Retirement is usually the time to enjoy life and realize longstanding dreams of youth. Psychodynamic models also explain creativity in PD, as a need to sublimate the accumulation of all the large and small frustrations of living with PD every day: inability to carry out plans, being made fun of in public, being unable to wear the same clothes anymore, no longer being able to perform certain dance steps, being discouraged from driving a car. Art is the expression of the soul and the PD patient’s soul has a lot to express.

### Limits

Our work provides the first group study linking creativity to dopamine agonists’ therapy in PD. However, the present study has several methodological and conceptual limits. The sample sizes are relatively small, and can question the generalization of our results to PD candidates to surgery and to PD in general. However, we present convincing statistically significant results on this small population of selected patients to surgery. We propose to generalize our results to PD patients without relevant executive dysfunction (as it is the case in patients selected for STN DBS) and exposed to dopamine agonists. In spite of PD, these patients conserve good cortico-cortical projection functioning. We do not intend to generalize our results to cognitively impaired PD patients, with important spread of alpha-synucleinopathy in the different cortical areas (51). Even if exposed to dopamine agonists, these cognitively impaired patients might not develop creativity, because they are lacking the basis of creative acts: concept elaboration, fluency, mental flexibility, i.e., intact executive function (52). Furthermore, creativity was evaluated on a single item on a single scale. However, no specific validated tool does exist in the context of PD. The existing psychometric divergent thinking battery that was used in PD, the Torrance Test of Creative Thinking did not permit to differentiate creative PD patients from healthy controls without creativity, which questions its validity (48).

## Conclusion

Creative patients have higher doses of dopamine agonists than controls presenting the same motor severity. Creativity appears with, or is exacerbated by, dopaminergic treatment, and decreases when dopaminergic treatment is reduced, in the context of STN stimulation. Dopamine agonists have a more selective affinity with D3 dopaminergic receptors, which are more highly represented in the mesolimbic system, than l-DOPA which has a more diffuse and balanced action on all dopaminergic receptors. Overstimulation of mesolimbic dopaminergic systems seems to facilitate the drive to create in PD patients who possess a certain “creative intelligence,” in a non-specific way, via the enhancement of all directed behaviors with a reward component. Dopaminergic treatment in PD induces ultra-rapid mood cycles, with euphoric/dysphoric oscillations, constituting a favorable ground for creativity, as it is the case in bipolar disorder. While behavioral addictions and impulse control disorders are well-known potentially devastating side effects, creativity can generally be considered as a highly beneficial side effect of dopamine agonists.

## Author Contributions

Eugénie Lhommée and Alina Batir: substantial contributions to conception and design, acquisition of data, analysis and interpretation of data, drafting the article, final approval of the version to be published. Jean-Louis Quesada: substantial contributions to conception and design, analysis and interpretation of data, revising it critically for important intellectual content, final approval of the version to be published. Claire Ardouin: substantial contributions to conception and design, acquisition of data, analysis and interpretation of data, revising it critically for important intellectual content, final approval of the version to be published. Valérie Fraix, Eric Seigneuret, Stéphan Chabardès, and Alim-Louis Benabid: acquisition of data, revising it critically for important intellectual content, final approval of the version to be published. Pierre Pollak: substantial contributions to conception and design, acquisition of data, revising it critically for important intellectual content, final approval of the version to be published. Paul Krack: substantial contributions to conception and design, acquisition of data, analysis and interpretation of data, drafting the article, revising it critically for important intellectual content, final approval of the version to be published.

## Conflict of Interest Statement

Eugénie Lhommée: none, Alina Batir: none, Jean-Louis Quesada: none, Claire Ardouin: none, Valérie Fraix: none related to the topic of the paper, Eric Seigneuret: none, Stéphan Chabardès: (1) preclinical research grants from Medtronic, not related to the topic of the paper and (2) travel reimbursements, Alim-Louis Benabid: none related to the topic of the paper, Pierre Pollak: none related to the topic of the paper, Paul Krack: (1) grants: GSK, Eutherapie, Novartis, UCB, Boehringer Ingelheim, Orkyn, Aguettant, Teva, Lundbeck, Medtronic, St Jude; (2) advisory boards: Novartis Abbott.
